# Positive regulation of prostate cancer cell growth by lipid droplet forming and processing enzymes DGAT1 and ABHD5

**DOI:** 10.1186/s12885-017-3589-6

**Published:** 2017-09-06

**Authors:** Ranjana Mitra, Thuc T. Le, Priyatham Gorjala, Oscar B. Goodman Jr.

**Affiliations:** 1 College of Medicine, Roseman University of Health Sciences, 10530 Discovery drive, Las Vegas, NV 89135 USA; 2 Comprehensive Cancer Centers of Nevada, 9280 W Sunset Road, Las Vegas, NV 89148 USA

**Keywords:** DGAT1, ABHD5, Lipid signaling in neoplastic cells

## Abstract

**Background:**

Neoplastic cells proliferate rapidly and obtain requisite building blocks by reprogramming metabolic pathways that favor growth. Previously, we observed that prostate cancer cells uptake and store lipids in the form of lipid droplets, providing building blocks for membrane synthesis, to facilitate proliferation and growth. Mechanisms of lipid uptake, lipid droplet dynamics and their contribution to cancer growth have yet to be defined. This work is focused on elucidating the prostate cancer-specific modifications in lipid storage pathways so that these modified gene products can be identified and therapeutically targeted.

**Methods:**

To identify genes that promote lipid droplet formation and storage, the expression profiles of candidate genes were assessed and compared between peripheral blood mononuclear cells and prostate cancer cells. Subsequently, differentially expressed genes were inhibited and growth assays performed to elucidate their role in the growth of the cancer cells. Cell cycle, apoptosis and autophagy assays were performed to ascertain the mechanism of growth inhibition.

**Results:**

Our results indicate that DGAT1, ABHD5, ACAT1 and ATGL are overexpressed in prostate cancer cells compared to PBMCs and of these overexpressed genes, DGAT1 and ABHD5 aid in the growth of the prostate cancer cells. Blocking the expression of both DGAT1 and ABHD5 results in inhibition of growth, cell cycle block and cell death. DGAT1 siRNA treatment inhibits lipid droplet formation and leads to autophagy where as ABHD5 siRNA treatment promotes accumulation of lipid droplets and leads to apoptosis. Both the siRNA treatments reduce AMPK phosphorylation, a key regulator of lipid metabolism. While DGAT1 siRNA reduces phosphorylation of ACC, the rate limiting enzyme in de novo fat synthesis and triggers phosphorylation of raptor and ULK-1 inducing autophagy and cell death, ABHD5 siRNA decreases P70S6 phosphorylation, leading to PARP cleavage, apoptosis and cell death. Interestingly, DGAT-1 is involved in the synthesis of triacylglycerol where as ABHD5 is a hydrolase and participates in the fatty acid oxidation process, yet inhibition of both enzymes similarly promotes prostate cancer cell death.

**Conclusion:**

Inhibition of either DGAT1 or ABHD5 leads to prostate cancer cell death. Both DGAT1 and ABHD5 can be selectively targeted to block prostate cancer cell growth.

## Background

Cancer is characterized by dysregulated growth and proliferation; in proliferating malignant cells there is an enhanced requirement for building blocks, including amino acids, nucleic acids and lipids. In addition to modulating glucose metabolism and energy production [1, 2], neoplastic cells also alter lipid metabolic pathways [3, 4] factoring net biosynthesis over energy production [5]. In various cancers, lipogenesis and cholesterol synthesis pathways are upregulated and several of these over expressed genes correlate with poor prognosis [6, 7]. In contrast to carbohydrate metabolism, little is known about the role of fatty acid metabolism in promoting cancer cell growth and metastasis [8, 9].

Recent studies have shown that cancer cells not only use fatty acids as a building blocks but also use them preferentially for ATP production through fatty acid oxidation [10, 11]. Neoplastic cells alter lipid metabolizing enzymes, triggering oncogenic signaling to promote growth [12]. Dysregulated lipid metabolism also promotes aberrant cancer cell-stromal cell communication, contributing to disease progression. In some cancer types, neoplastic cells derive energy from supporting host cells by modulating their metabolic activity [13, 14]. In several cancers dysregulated fatty acid (FA) synthesis, storage, uptake transport and degradation are associated with disease outcome. Some of these cancer cells are known to upregulate FA synthesis which in turn supports rapid proliferation and decreased drug sensitivity [12, 13, 15, 16]. Cancer cells tend to alter FA synthesis by increasing production of fatty acid precursors glutamine and citrate; alternately they also uptake extracellular FA for use as building blocks, energy production and storage [17–19]. Knockdown studies on FA synthesis genes show poor prognosis and decreased overall survival in several cancers including prostate [13, 18, 20, 21] hence FA synthesis genes have been implicated as therapeutic targets [15].

Our recent studies demonstrate that cancer cells tend to uptake FA and store them as lipid droplets which can be used later to aid proliferation [17, 22–24]. The preferential uptake of lipids over glucose in prostate cancer circulating tumor cells has been assessed for potential therapeutic targeting [25]. Upon entering the circulation, CTCs uptake lipid, storing them in the form of lipid droplets that may be used subsequently for growth and proliferation at the metastatic site. As the neoplastic cells uptake increasing amount of FA, size and number of the lipid droplets increase [26]. The increase of lipid droplet size is an indication of increased TG mass which is catalyzed by several enzymes present on the lipid droplet monolayer in collaboration with ER which plays a major role in lipid droplet dynamics [27, 28].

The enzymes involved in the synthesis of TG from FA aid in the increase of size and number of lipid droplets whereas lipolysis enzymes metabolize TG for energy production and membrane synthesis for cell proliferation. The major enzymes involved in TG synthesis and storage are diglyceride acyltransferase (DGAT), monoacylglycerol acyltransferase (MGAT), glycerol-3-phosphate acyltransferase (GPAT) and enzymes involved in cholesterol metabolism like ACAT (acyl-CoA cholesterol acyl transferase) [29–32]. Enzymes involved in lipolysis include hormone sensitive lipase (HSL), monoacylglycerol lipase (MGL) and adipose triglyceride lipase (ATGL). Additionally there are set of lipid droplet associated proteins which regulate cellular lipid stores namely-perilipin, adipose fatty acid binding protein, caveolin, alpha/beta- hydrolase domain containing protein 5(ABHD5), tail interacting protein 47 (TIP47), OXPAT and fat-specific protein (FSP27) [29, 31, 33]. All the above mentioned proteins regulate and maintain a balance between TG synthesis and lysis eventually deciding the size and number of fatty acid droplets in the cells. Based on the observation that neoplastic cells show increased accumulation of large lipid droplets we hypothesize that this results by controlling the balance of the above mentioned proteins.

The focus of this study is to understand the differences in FA uptake and storage between malignant epithelial cells versus peripheral blood mononuclear cells (PBMCs), with the broader goal of identifying and therapeutically targeting the tumor-specific metabolic changes. As fatty acids in malignant epithelial cells are generally stored as lipid droplets, we analyzed differential gene expression relevant to lipid droplet formation and processing between PBMCs and LNCaP cells. We then analyzed the individual differentially expressed genes in prostate cancer cell growth.

## Methods

### Cell lines, kits and antibodies

LNCaP (CRL-1740), HeLa (CCL-2) and OP9 (CRL-2749) cells were purchased from ATCC (Manassas, VA) and maintained in RPMI, MEM and alpha MEM media respectively from Invitrogen, (Carlsbad, CA). Differentiation in OP9 cells is induced by replacing MEM alpha medium with knockout serum at 15% concentration. PBMCs were isolated from fresh volunteer blood samples or were purchased along with plasma from Bioreclamation IVT, USA. PBMCs were growth in X-vivo15 media with or without phenol red (for MTS assay) from Lonza (Allandale, NJ). The siRNA transfection reagents, oligofectamine and OPTIMEM were from Invitrogen; the siRNAs were from Dharmacon (Thermo scientific). DGAT1 (diglyceride acyltransferase, ab181180), ABHD5 (alpha/beta- hydrolase domain containing protein 5, ab183739), ATGL (adipose triglyceride lipase, ab99532) and ACAT (Acyl-CoA cholesterol acyl transferase, ab168342) antibodies were from Abcam (Cambridge, MA). Cyclin antibodies (cyclin antibody sampler kit #9869S), ACC (acetyl CoA carboxylase) and AMPK (AMP activated protein kinase) antibodies (AMPK and ACC sampler kit #9957S), raptor (regulatory-associated protein of mTOR) and ULK (serine/threonine-protein kinase) antibodies (ULK1 sampler kit #8359S), LC3B (microtubule-associated protein 1A/1B–light chain 3) antibody (autophagosome marker antibody sampler kit #8666 s) PARP (Poly ADP-ribose polymerase, #9542) and pP70S6/P70S6 (ribosomal protein S6 kinase, #9205 and #2708) were from Cell Signaling Technology (Danvers, MA). GAPDH antibody was from Fitzgerald (Acton, MA). MTT, crystal violet, oil red and propidium iodide was from Sigma-Aldrich (St. Louis, MO). The secondary antibodies were from LI-COR (Lincoln, NE). MTS cell titer reagent was from Promega (Madison,WI). CellLite BacMam 2.0 lysosome-RFP and bodipy dye was from Molecular Probes, (part of Thermo Fisher Scientific, San Deigo, CA). Ficoll paque (density 1.077) was from GE health care (Milwaukee, WI). Apoptosis/ necrosis detection kit was from Abcam (Cambridge, MA).

### Western blotting

Cells were lysed using RIPA buffer supplemented with protease and phosphatase inhibitors after the desired treatment was performed. GAPDH was used as an internal control. Infrared fluorescent-labeled secondary antibodies (IR dye 680 and IR dye 800) from LICOR Biosciences (Lincoln, NE) were used for detection using Odyssey CLx, which reduces background substantially. The bands were quantified using Odyssey software, which calculates pixel density and automatically takes an area adjacent to each band for background corrections.

### siRNA inhibition

Cells were plated in complete media without antibiotics on a poly D-lysine-coated plate (80,000 cells per 6 well). After 48 h. of growth the cells were transfected using RNAimax according to the manufacturer’s instructions. The smart pool non-target (NT) siRNA was used as a transfection control with the experimental target gene siRNAs. A pool of four siRNA against the target genes were used to block the expression. The final concentration of the siRNA (NT and targets) was 30 nM. For cell growth assays, cells were trypsinized 24 h after transfection and replated in 96 well plates (2000 cells/well).

### Growth assay

Growth of the cells was measured using MTT assays and/or clonogenic assays. For MTT (3-(4,5-Dimethylthiazol-2-yl)-2,5-Diphenyltetrazolium bromide) assay, 2000 cells/ 96 well or 13,000 cells/ 24 well were plated then treated for required time periods as mentioned and incubated with MTT (4 mg/ml) for 2 h. at 37 °C. Cells were centrifuged at 2000 g for 10 min and the supernatant was discarded. The cell pellet was dissolved in 100/500 μl of DMSO. A plate reader was used to read the absorption at 540 nm. Experiments were performed in octuplet/quadruplet and repeated at least three times. For clonogenic assay, cells were trysinized 48 h after siRNA transfection and dilution (1:500 and 1:1000) plated in 6 well plates. After 2 to 3 weeks, the colonies were fixed, stained (0.75% crystal violet, 50% ethanol and 1.75% formaldehyde) and counted. The assay was repeated three times.

### Lipid staining assays

Lipids were stained using either oil red O (ORO) or bodipy. For staining cells with ORO they were washed with DPBS and fixed in 10% formaldehyde for 30 min at RT. Prior to staining a stock solution of 3 mg/ml was prepared in isopropanol. The stock solution was diluted in water (3:2) and incubated at room temperature for 10 min to prepare the working solution. The working solution was filtered using Whatman filter prior to staining the cells. The fixed cells were washed with water, and then incubated with 60% isopropanol for 5 min. After this the filtered oil red working solution was added for 5 min and then washed with water before viewing under the microscope. For staining with bodipy the cells didn’t need to be fixed, as the dye is cell permeable. For staining with bodipy cells were washed with 0.1% BSA in DPBS. The stock of bodipy dye was also prepared in the same solution. The cells were stained in bodipy solution (0.5 μg/ml, final) for 5 min and then again washed with DPBS containing BSA. Cells were visualized under the fluorescent/ confocal microscope in the FITC channel.

### Cell cycle analysis

Cell cycle analysis was performed after staining the cells with propidium iodide (PI). The cells were trypsinized washed with PBS and fixed in 70% ethanol (optional) and then stained with PI in nicoletti buffer (propidium iodide 50 μg/ml, 0.1% sodium citrate, 0.1% triton X-100, RNase 1 mg/ml, in DPBS). Cells were analyzed using C6 Accuri flow cytometer (Becton Dickinson, Mountainview, CA). ModFit LT software was used to analyze and assign percentage values to cells present in the different cell cycle stages and remove the debris and aggregates.

### Apoptosis assay

To test apoptosis; apoptosis/necrosis detection kit from Abcam (Cambridge, MA) was used. The kit uses apopxin green indicator dye which binds to phosphatidylserine which is flipped outside during apoptosis. 7-AAD dye in the kit is able to detect late apoptotic and necrotic cells as it is a membrane impermeable dye and stains cells with loss of plasma integrity. Cells were trypsinized 72 h after the siRNA treatment and stained with apopxin and 7-AAD for 30 min at RT in assay buffer as described in the manufacturers protocol. The cells were analyzed using C6 Accuri flow cytometer. The apopxin dye is generally read in FL1 channel (Ex/Em = 490/525) whereas 7-AAD is read in the FL3 channel (Ex/Em = 550/650). Alternatively cells were grown on glass plates and after staining with apopxin and 7-AAD were analyzed using the Nikon Elipse Ti confocal microscope.

### Autophagy detection assays

We looked at prevalence of lysosomes for detection of autophagy and confirmed it with western blotting of LC3BII (microtubule-associated protein 1A/1B–light chain 3) and immunocytochemistry studies. For lysosomal detection we used cell light reagent BacMam 2.0 (RFP), it is a baculovirus based fluorescent protein-signal peptide fusion targeting lysosomes. The transduction method was followed as recommended by the manufacturer. Cells (40,000) were plated in the glass bottom petri plates, after 48 h cells were transduced with the reagent (24 μl). Cells were visualized under confocal microscope after overnight incubation with the reagent. Western blotting procedure using LC3B antibody was performed as described earlier. The ratio (less than one) of band intensity of LC3BI and II (faster) was used to confirm autophagy. LC3B antibody was also used in an immunocytochemistry study and intense punctate staining confirmed autophagy.

### Immunofluorescence staining and analysis

Cells were either plated on glass bottom petriplates or were transferred to glass slides by cytospin after the appropriate specified treatment. The cells were fixed in 3.7% paraformaldehyde for 10 min followed by permeabilization for 5 min using 0.2% Triton X-100 in PBS. The cell were then washed twice and blocked with 10% goat serum and 1% BSA in PBS for 30 min at 37 °C followed by staining with the primary antibody (1:100 dilution, 1% BSA in PBS) for 2 h at 37 °C. After the incubation, the cells were washed three times (5 min) with PBS and incubated with secondary-TRITC labelled antibody for 1 h at 37 °C, washed three times with PBS, and mounted with DAPI (40,6-Diamidino-2-phenylindole dihydrochloride) for nuclear staining. Cells were visualized under a Nikon Elipse Ti confocal microscope and captured using NIS-elements imaging software. The experiments were repeated three to four times and the figures are representative maximum image projections with the same laser power settings.

## Results

### Screening of differentially expressed lipid droplet forming and processing genes in cancer cells

**Fig. 1:**
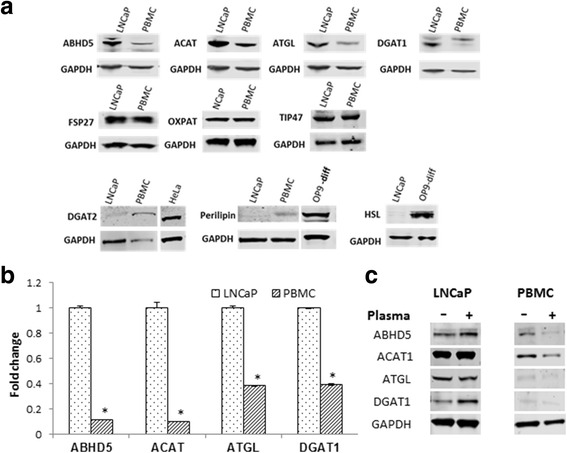
Comparative expression of fatty acid metabolism regulating genes in LNCaP and PBMCs. **a** Screening for fatty acid metabolism genes which are differentially expressed in LNCaP and PBMCs was done using western blot analysis; the genes differentially expressed are shown. GAPDH is used as an internal control. The experiment was repeated three times and a representative experiment is shown. HeLa and differentiated OP9 cell extracts have been used as positive control for DGAT2, perilipin and HSL expression. **b** Histogram plot showing comparative fold expressions of ABHD, ACAT, ATGL and DGAT1 between LNCaP and PBMCs. The fold change is calculated by taking base levels expressed in LNCaP cell line as 1 after GAPDH calibration and is an average of three separate experiments, (*)-(*P* ≤ 0.05). **c** Effect of plasma treatment on the expression levels of ABHD5, ACAT1, ATGL and ABHD5. Western blots were performed after 50% plasma treatment for 12 h. on both PBMCs and LNCaP cells. Out of the three experimental repeats a representative experiment is shown. Fold changes mentioned in the results section are an average of all the experiments

As circulating tumor cells (CTCs) and malignant epithelial cells uptake and accumulate fatty acid once in circulation or in presence of plasma [17, 34]. We therefore hypothesize that genes involved in fatty acid storage are differentially expressed in malignant cells versus PBMCs. We screened and compared the expression pattern of 10 genes involved in lipid droplet formation and processing in LNCaP and PBMC incubated with and without plasma: (ABHD5, ACAT1, ATGL, DGAT1/2, FSP27, HSL, OXPAT, perilipin and TIP47) (Fig. 1a). The expression levels of each individual gene (fold change) were compared between LNCaP and PBMCs taking the expression levels in LNCaP as reference (one). Four genes ABHD5, ACAT1, ATGL and DGAT1 were overexpressed in LNCaP as compared to PBMCs. Expression of OXPAT, FSP27 and TIP47 were similar in both PBMCs and LNCaP cells, whereas PBMCs expressed higher levels of DGAT2 and perilipin. HSL was expressed in very low amounts and was almost undetectable by western in LNCaP cells (Fig. 1a). Western analysis showed that ABHD5, ACAT1, ATGL and DGAT1 were expressed 8.8, 10, 2.5 and 2.55 fold more respectively in LNCaP vs PBMCs (Fig. 1b). We further analyzed the effect of plasma incubation on the four genes (ABHD5, ACAT1, ATGL and DGAT1) overexpressed in LNCaP cells. In presence of plasma DGAT1 and ABHD5 expression increased by 2.5 and 2.0 fold respectively (*P* ≤ 0.05) in LNCaP, ATGL was down regulated (0.5 fold, *P* ≤ 0.05) and ACAT1 and did not show significant changes. On the other hand plasma incubation downregulated all the four differentially expressed genes DGAT1, ABHD5, ATGL and ACAT1 by 0.5, 0.2, 0.5 and 0.5 fold (*P* ≤ 0.05) respectively in PBMCs (Fig. 1c).

### Effect of inhibition of ABHD5, ACAT1, ATGL and DGAT1 on growth of LNCaP cells

**Fig. 2:**
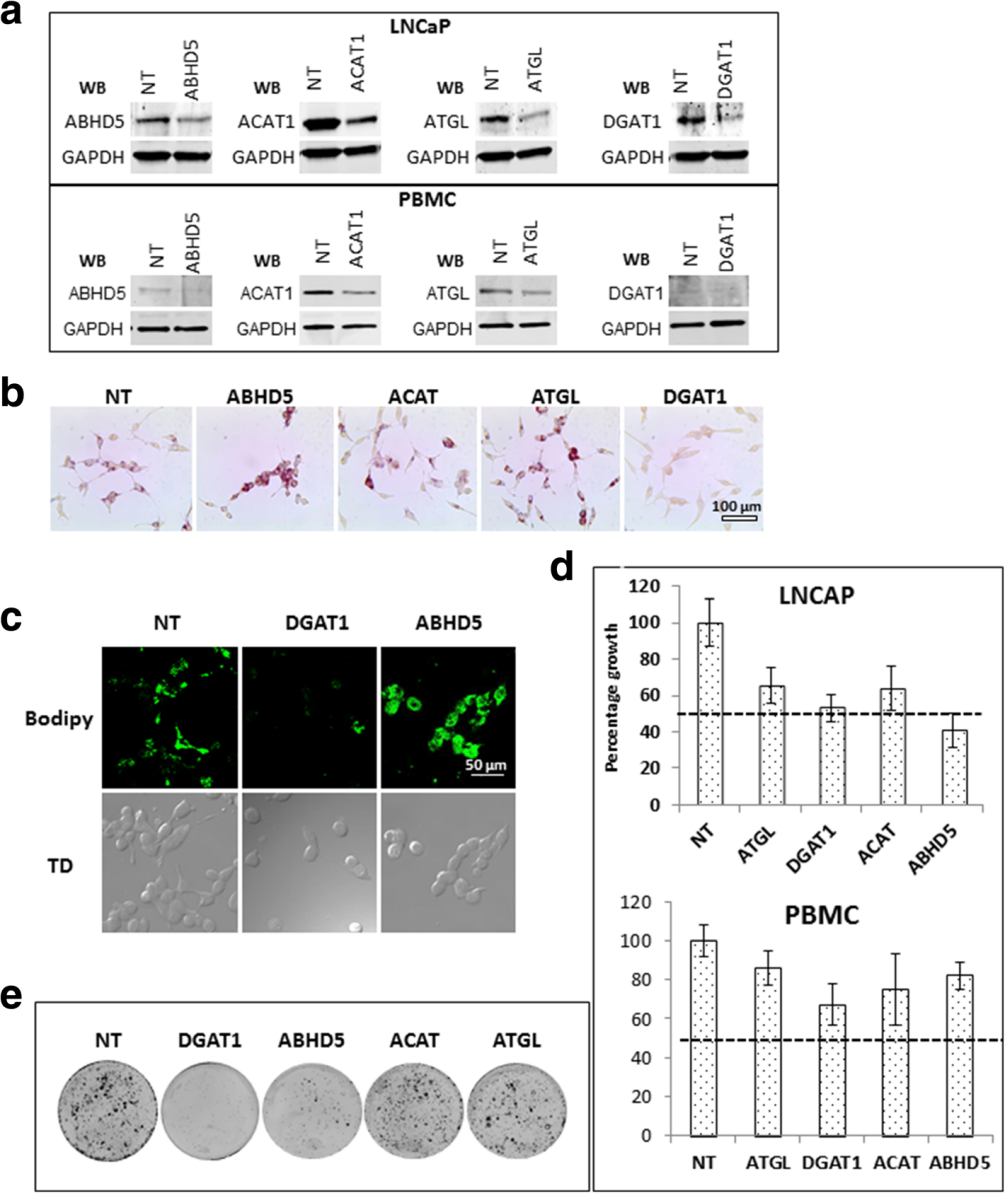
Effect of depletion of ABHD5, DGAT1, ACAT1 and ATGL on LNCaP cells: **a** western blot analysis 72 h after transfection with target and nontarget (NT) siRNA, the siRNA treatments are written on top of the blots and western blot (WB) primary antibodies used are mentioned on the left of each blot. **b** and **c** Oil red and bodipy staining performed 72 h after the siRNA transfection, the specific siRNA treatments are mentioned on top of the figures. **d** An MTS based assay showing relative growth 72 h after transfection with respective siRNAs. Assay was done in octuplets, the dotted line represents 50% growth inhibition. **e** Clonogenic assay showing number of colonies 16 days after respective siRNA transfection. All the assays were performed at least three times, representative experiments have been shown

To study the role of the genes overexpressed in LNCaP cells we blocked the individual genes using siRNA and with non-target (NT) siRNA as control. We obtained approximately 30–60% inhibition of the protein expression of the targeted genes in LNCaP (Fig. 2a). We also simultaneously inhibited the genes in PBMCs using siRNA; despite low transfection efficiency in these we observed significant inhibition of the target genes (Fig. 2a). The functional loss of gene expression was monitored using ORO and bodipy stains in LNCaP. As anticipated inhibition of ABHD5 and ATGL show increased lipid accumulation where as DGAT1 inhibition resulted in loss of lipid droplet formation (Fig. 2b, c). We used a MTS assay to monitor the growth after siRNA inhibition 72 and 96 h after transfection. Our results indicate that although all the four genes can inhibit LNCaP cell growth, ABHD5 and DGAT1 do so most potently (45–60% growth inhibition) (Fig. 2d). All the four targeted genes resulted in considerably less (15–25%) growth inhibition in PBMCs, detectable using a specialized growth media (X-vivo15) for PBMCs which promotes growth (30% growth after 72 h) (Fig. 2d). As both the expression and the growth inhibition of PBMCs upon silencing of these genes were minimal, we focused on the LNCaP for subsequent experiments. Clonogenic assay results which shows long term effect on growth further showed that ABHD5 and DGAT1 siRNA prevented the formation of 90–95% of colonies as compared to NT control (Fig. 2e). Since we observed maximum inhibition of growth with ABHD5 and DGAT1 siRNA we examined the mechanism by which these two genes promote prostate cancer growth.

### ABHD5 and DGAT1 siRNA cause G0/G1 cell cycle block in LNCaP cells

**Fig. 3:**
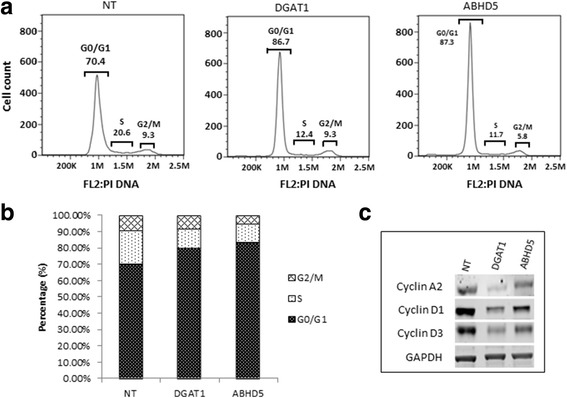
Effect of DGAT1 and ABHD5 siRNA on cell cycle: **a** Cell cycle analysis showing G0/G1 block 72 h after siRNA treatment with NT, DGAT1 and ABHD5 siRNA. **b** Graphical representation showing percentage of cells in each phase of cell cycle with siRNA treatment. **c** Western analysis of cyclins involved at the G1/S check point 72 h after siRNA treatment. GAPDH has been used as an internal control. Experiments have been performed three times and representative experiment has been shown

Cell cycle assays were performed 72 h. after siRNA inhibition as described in materials and method. Both DGAT1 and ABHD5 siRNA resulted in approximately ~17% increase in G0/G1 cell population as compared to NT (Fig. 3a and b). This increase in the G0/G1 cells correlated with downregulation of cyclin A2, cyclin D1 and cyclin D3 known to play important role at G0/G1 check point, ranging 0.2–0.35 fold and 0.3–0.55 fold with DGAT1 and ABHD5 siRNA treatment respectively, *P* ≤ 0.05.(Fig. 3c).

### ABHD5 siRNA causes apoptosis whereas DGAT1 siRNA causes autophagy in LNCaP cells

**Fig. 4:**
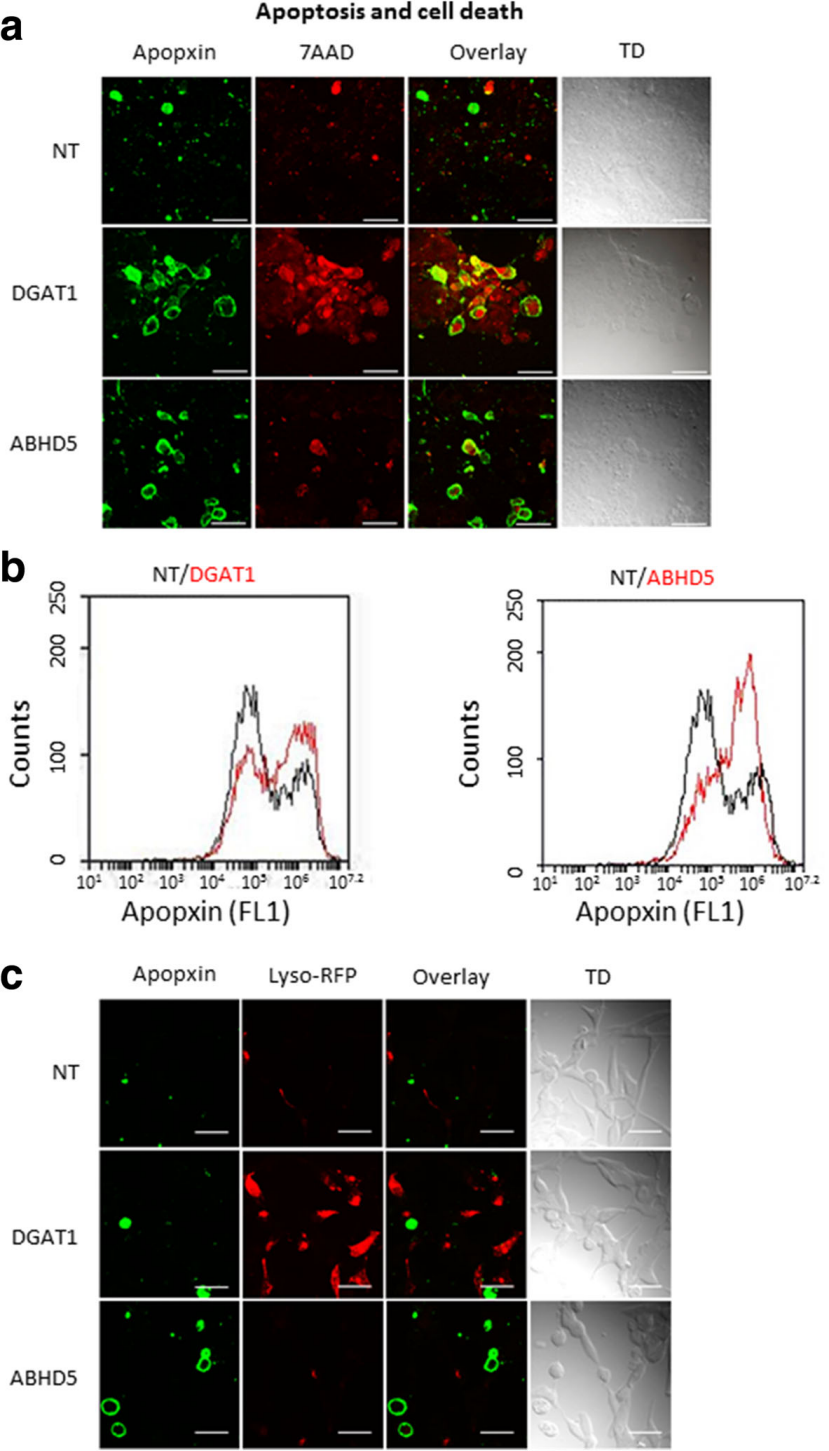
Blocking of ABHD5 and DGAT induces apoptosis and autophagy respectively in LNCaP cells: **a** Immunofluorescent image showing staining with apopxin (green) and 7AAD (red) after siRNA treatment (72 h). Green only-early apoptotic cells, red only-dead cells and red and green both - late apoptotic or cells with damaged plasma membrane. **b** Apoptosis assay showing increased cell population of apopxin positive cells with ABHD5 siRNA as compared to NT siRNA using flow method. Black-NT siRNA, Red- DGAT1 and ABHD5 siRNA. Apopxin peak (x axis) at 10^6^ represents the apoptotic cell population. **c** Immunofluorescent image showing staining with apopxin (green) and Lyso tracker (red) after siRNA treatment (72 h). All immunofluorescent experiments have been performed three times and a representative experiment has been shown. For each experiment 4–5 xy planes were randomly selected for microscopy. Maximum intensity image has been shown in the figures which either included all the z-stacks (**a**) or some selected z-stack (**c** - apopxin staining) to depict localization. Bar is 50 μm

Next we examined apoptotic and autophagic cell death pathways involved in this process. We assessed apoptosis after NT, ABHD and DGAT siRNA treatment using apopxin and 7AAD to differentiate between non-viable (7-AAD positive), early apoptotic (only Apopxin positive), and late apoptotic (positive for both) cells. Fluorescent microscopy of these cells revealed a mix of early (only green) late apoptotic (both red and green) and dead cells (only red) with ABHD inhibition (Fig. 4b). In contrast DGAT siRNA we mostly observed double stained cells positive for both 7AAD and apopxin or 7AAD alone (Fig. 4a). To understand the reasoning behind lack of early apoptotic cells with DGAT siRNA we performed flow cytometric analysis after apopxin staining. The flow analysis reveals two cell populations: one with less apopxin staining (FL1–10^4^, healthy cells) and the other population with intense apopxin staining (FL1–10^6^), representing the actual apoptotic population. The LNCaP cells demonstrated increased apoptosis after ABHD5 siRNA treatment (Fig. 4b); on the other hand no such apoptotic population was observed after DGAT1 siRNA transfection. Given that cell death may occur by nonapoptotic mechanisms, as suggested by a lack of early apoptotic cells throughout the time course, we tested the possibility of autophagy in case of DGAT siRNA treatment. Staining with baculovirus-based lysosomal tracker dye revealed that while DGAT siRNA treatment generated lysosome-positive cells, NT or ABHD siRNA treatment (Fig. 4c) did not. Extensive lysosomal production in the cell is consistent with autophagic cell death, as observed in case of DGAT siRNA treatment.

### DGAT and ABHD5 inhibition modulate the AMPK pathway

**Fig. 5:**
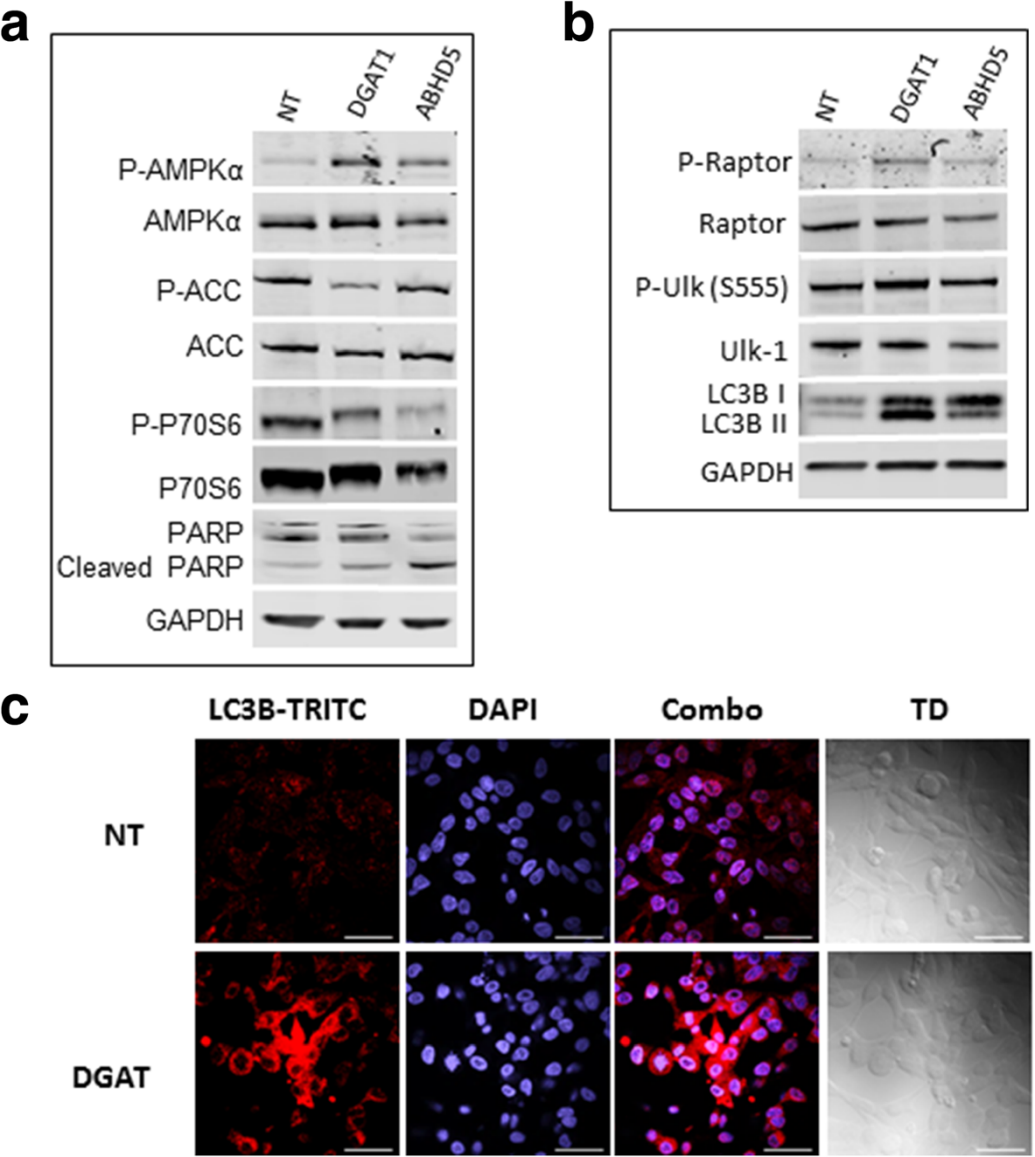
Immunoblotting and immunofluorescence experiments depicting pathways after ABHD5 and DGAT1 siRNA treatment leading to apoptosis and autophagy respectively: **a** and **b-** Immunoblotting assay using different antibodies after siRNA transfection with NT (non-target), DGAT1 and ABHD5 siRNA. GAPDH has been used as an internal control. A representative experiment is shown out of the three repeats. **c** Immunofluorescence assay showing LC3 punctate staining after DGAT siRNA treatment representing autophagy. DAPI (blue)-nucleus and TRITC (red)-LC3. Bar is 50 μm

As AMP-activated protein kinase (AMPK) plays a key role in cellular energy homeostasis regulating both glucose and fatty acid metabolism, we decided to assess whether inhibition of ABHD5 or DGAT1 perturbs fatty acid metabolism by the AMPK pathway. Our results indicate that inhibition of both DGAT1 and ABHD5 using siRNA resulted in increased phosphorylation of AMPKα (AMP activated protein kinase) (5.5 and 3.0 fold respectively, *P* ≤ 0.05) (Fig. 5a). Since increased phosphorylation of AMPK (T-172) can inactivate ACC by phosphorylating at (Ser79) and blocking malonyl-CoA production, we examined the downstream impact on ACC (acetyl CoA carboxylase) phosphorylation. DGAT1 siRNA blocked the phosphorylation of ACC (downregulated 0.25 fold, *P* ≤ 0.05) (Fig. 5a) indicating reduction in malonyl-CoA production. As malonyl-CoA is known to inhibit CPT1, a critical molecule involved in initiating fatty acid oxidation, lower malonyl-CoA production may lead to upregulation of CPT1 activity and increased fatty acid oxidation [35]. ABHD5 siRNA had no effect on ACC phosphorylation.

**Fig. 6:**
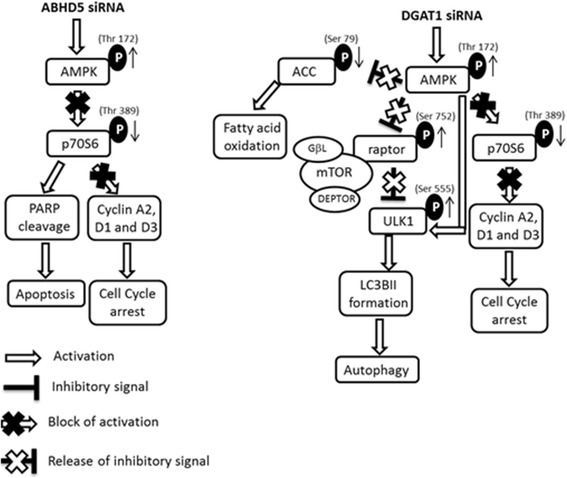
Schematic representation of possible apoptosis and autophagy triggering pathways after ABHD5 and DGAT1 siRNA treatment respectively. ABHD5 (alpha/beta- hydrolase domain containing protein 5), DGAT1 (diglyceride acyltransferase), AMPK (AMP activated protein kinase), ACC (acetyl CoA carboxylase), pP70S6/P70S6 (ribosomal protein S6 kinase), PARP (Poly ADP-ribose polymerase), raptor (regulatory-associated protein of mTOR), ULK (serine/threonine-protein kinase) and LC3B (microtubule-associated protein 1A/1B–light chain 3)

Consistent with our earlier findings that either siRNA treatment leads to increased G0/G1 cell population, we observed that phosphorylation of P70S6 (Thr 389, Ribosomal protein S6 kinase beta) was decreased 0.7 fold following DGAT1 siRNA treatment versus 0.27 fold with ABHD5 siRNA (Figs. 3a&b 5a, *P* ≤ 0.05). Reduction in P70S6 phosphorylation is known to triggers apoptosis, which was further supported by increased PARP cleavage (poly ADP ribose polymerase) with ABHD5 siRNA treatment indicating apoptotic signaling (Fig. 5a). On the other hand, DGAT1 siRNA increased raptor (regulatory-associated protein of mTOR) phosphorylation by 2.5 fold at Ser 752 which is known to release the inhibitory signal of mTOR on ULK-1 (serine/threonine-protein kinase) pathway initiating autophagy (Fig. 5b). Increased downstream P-ULK (ser555) phosphorylation was observed with DGAT1 siRNA treatment as expected (2 fold, Fig. 5b). Autophagic signaling after DGAT1 siRNA was further confirmed by increased ratio of LC3bII/LC3bI (microtubule-associated protein 1A/1B–light chain 3) band on western blotting which increased from 0.27 to 1.6 compared with NT siRNA control (Fig. 5b) and condensed punctate staining with immunofluorescence (LC3-TRITC) after DGAT1 siRNA treatment as compared to NT siRNA control (Fig. 5c). A schematic diagram representing the possible mechanisms by which DGAT1 and ABHD5 siRNA treatments decrease growth in prostate cancer cells by modulating lipid storage and processing pathways is shown in Fig. 6.

## Discussion

Cancer cells uptake FA rapidly and store them as lipid droplets suggesting that these cells have modified gene expression leading to upregulation of lipid droplet formation. Lipid droplets can be used to facilitate cancer cell growth by providing precursors essential for cell proliferation and/or by FA oxidization for energy production. Our results indicate that four enzymes ACAT, ATGL ABHD5 and DGAT1 are differentially overexpressed in prostate cancer cells as compared to PBMCs. These differentially expressed genes are involved both in the formation of the lipid droplets and in the processing of the FAs. ACAT is involved in the esterification of cholesterol and is known to promote accumulation of cholesterol ester in fat droplets. ATGL on the other hand is a lipase and is involved in the degradation of triglyceride (TG), consistent with our observation that ATGL siRNA leads to accumulation of lipid droplets. That we do not observe any effect of ACAT inhibition on cell growth suggests lipid accumulation in cancer cells is a cholesterol-independent process. As inhibition of both ACAT and ATGL did not affect the growth of the LNCaP cells we focused on the other two differentially identified genes DGAT1 and ABHD5 which effected growth. Whereas DGAT1 is involved in the synthesis of triacylglycerol, ABHD5 is involved in the degradation of triglycerides, similar to ATGL, and is known to be responsible for maintaining lipid homeostasis [36]. Consistent with these mechanisms of action we observe that DGAT1 siRNA leads to absence of TG formation and shows absence of lipid droplets where as ABHD5 siRNA leads to accumulation of lipid droplets. As ABHD5 activates ATGL a critical component in lipolysis initiation it is possible that the lipid accumulation observed with ABHD5 siRNA treatment is due to blocking of lipolysis initiation. ATGL siRNA treatment did not result in as much lipid droplet accumulation as ABHD5 siRNA treatment and did not affect the growth of LNCaP cells. This possibly may be because ABHD5 is regulating triacylglycerol metabolism independently of ATGL in LNCaP, similar to prior reports in hepatocytes [37]. ATGL-independent function of ABHD5 is reported but the mechanism not well understood [38–40].

In the process of lipid droplet formation DGAT1 and ABHD5 genes seem to maintain a balance between the FA storage and usage. Blocking of DGAT1 leads to blockage of storage while blockage of ABHD5 leads to blockage of usage; hence both the siRNA treatments lead to unavailability of FA for cancer cell proliferation and decreased growth (Fig. 2d and e). The seemingly opposing functions suggest that lipid droplet homeostasis is tightly regulated and may be modulated according the needs of the cancer cells.

Our results indicate that both the siRNA treatments promote a G0/G1 population increase resulting from slow passage of cells through the G1/S check point associated with decrease in cyclins A2, D1 and D3. Further, our results show that both DGAT1 and ABHD5 results in cell death, the mechanism of cell death is very different, one triggers autophagy and the other trigger apoptosis. ABHD5 siRNA shows an increase in AMPK phosphorylation (Thr 173) and decrease in P70S6 phosphorylation, decrease in P70S6 phosphorylation is known to result in increased phosphorylation of eEF2 and inhibition of protein synthesis [41–43] leading to apoptosis (cleaved PARP). From our experimental results we conclude that the cell death with ABHD5 inhibition involves AMPK/P70S6 axis leading to blockage of protein synthesis and apoptosis (Fig. 6). On the other hand although DGAT1 siRNA also increases AMPK phosphorylation it does not affect the P70S6/apoptosis pathway, instead it shows inhibition of ACC phosphorylation leading to reduced fatty acid synthesis and oxidation. Simultaneously inhibiting DGAT1 also leads to phosphorylation of raptor, inhibiting the m-TOR pathway and triggering autophagy [44, 45]. The initiation of autophagy correlates with increased phosphorylation of ULK1 (S555), both m-TOR inhibition and AMPK phosphorylation can directly phosphorylate ULK1 [46]. The triggering of autophagy and cell death is also further confirmed with LC3 punctate staining and the decrease in the ratio of LC3B I and II bands [47–49]. Collectively our data indicate that DGAT1 inhibition leads to decreased energy metabolism, and cell death by autophagy by inhibition of m-TOR pathway via AMPK/ACC and AMPK/raptor/ULK1 axis respectively (Fig. 6).

Our results strongly suggest that inhibition of both DGAT1 and ABHD5 promote prostate cancer cell death. Since both DGAT1 and ABHD5 are overexpressed in cancer cells and CTCs vigorously uptake lipid as compared to PBMCs, targeting them may represent a new oncolytic approach. Targeting lipogenic enzymes to block cancer growth have been the subject of several studies and their efficacy as anticancer agents have been proven [20, 50]. In colorectal cancer it has been shown that ABHD5 expressed in tumor associated macrophages promote cancer growth [51]. ABHD5 knockdown is lethal in animal models and ABHD5 deficient mice die within hours of being born. There are no known inhibitors of ABHD5 in development, likely due to these global off target effects. As ABHD5 is known to interact with perilipin and activate ATGL [52–54], another alternate approach can be to use small molecule ATGL inhibitors. ATGL deficient mice develop hepatic steatosis but are viable [39]. Our results show that LNCaP cells treated with ATGL siRNA do not accumulate as much lipid droplets as compared with ABHD5 siRNA treatment and do not cause as much cell death (Fig. 2). This may be due to alternate regulation of triacylglycerol metabolism by ABHD5, independently of ATGL. Thus targeting ABHD5 will further require understanding of this alternate function and presently limits a dual inhibition approach of both ABHD5 and DGAT1. However several DGAT1 inhibitors are being developed for treating obesity and other metabolic diseases [55, 56], and some of these are in clinical trials [57–59]. Utilizing these available small molecule DGAT1 inhibitors in future studies will help assess their therapeutic feasibility in prostate cancer.

## Conclusions

In summary our findings indicate that inhibition of both DGAT1 and ABHD5 using siRNA leads to reduction in prostate cancer cell growth. DGAT1 is known to aid in the formation of lipid droplets where as ABHD5 is involved in processing of these droplets. Inhibition of both DGAT1 and ABHD5 leads to cell cycle block and cell death either by triggering apoptosis (ABHD5 inhibition) or autophagy (DGAT1 inhibition). DGAT1 inhibition leads to autophagy through activation of raptor-ULK1-LC3 pathway. ABHD5 inhibition leads to downregulation of P70S6 phosphorylation blocking protein synthesis and triggering apoptosis through PARP cleavage. As prostate cancer cells overexpress DGAT1 and ABHD5; both can be targeted to block prostate cancer cell growth.

## Abbreviations

ABHD5: Alpha/beta- hydrolase domain containing protein 5
ACAT: Acyl-CoA cholesterol acyl transferase
ACC: Acetyl CoA carboxylase
AMPK: AMP activated protein kinase
ATGL: Adipose triglyceride lipase
DGAT1: Diglyceride acyltransferase
LC3B: Microtubule-associated protein 1A/1B–light chain 3
P70S6: Ribosomal protein S6 kinase
PARP: Poly ADP-ribose polymerase
Raptor: Regulatory-associated protein of mTOR
ULK: Serine/threonine-protein kinase
